# The Mediating Effects of Nutritional Status on the Relationship between Number of Residual Teeth and Cognitive Function among Older Adults: A Cross-Sectional Multicenter Study

**DOI:** 10.3390/nu15143089

**Published:** 2023-07-10

**Authors:** Yun Li, Xin Xia, Wenwen Wu, Xin Tian, Yuexia Hu, Birong Dong, Yanyan Wang

**Affiliations:** 1 West China School of Nursing, West China Hospital, Sichuan University, Chengdu 610041, China; yunliscu@stu.scu.edu.cn (Y.L.); yuexia.hu1996@gmail.com (Y.H.); 2 National Clinical Research Center for Geriatrics, West China Hospital, Sichuan University, Chengdu 610041, China; xiaxin@wchscu.cn; 3 West China School of Nursing, Innovation Center of Nursing Research, Nursing Key Laboratory of Sichuan Province, West China Hospital, Sichuan University, Chengdu 610041, China; 18781932712@163.com; 4 Nursing Key Laboratory of Sichuan Province, National Clinical Research Center for Geriatrics, Science and Technology Department, West China Hospital, Sichuan University, Chengdu 610041, China

**Keywords:** mediation analysis, dentition, cognition, nutrition disorders

## Abstract

The underlying mechanisms of the relationship between the number of teeth and cognition is still unclear. We aimed to construct a mediation model between the number of residual teeth and cognitive function, using nutritional status as a mediating factor. This study was completed using the West China Health and Aging Trend cohort. A total of 6634 multi-ethnic older adults, aged 50 years or older, were included. This study measured cognitive function using the Short-Portable Mental Status Questionnaire, and nutritional status was assessed using the Mini Nutritional Assessment-Short Form. The mediation analysis examined the potential mediating role of nutritional status. The pathway analysis was supplemented and validated using the structural equation modelling framework. Multiple linear regression demonstrated that a higher number of residual teeth was correlated with enhanced cognitive function (*β* = −0.15; 95% CI: −0.19 to −0.111). The mediation model, from the number of residual teeth to cognitive impairment, was partially mediated by nutritional status (*β* = −0.0608; 95% CI: −0.0762 to −0.0461). The proportion of the mediating effect, expressed as a percentage, was 40.66%. Furthermore, the estimated coefficients for the number of residual teeth and nutritional status varied across ethnic groups. This study indicated that enhancing the nutrition of older adults could reduce the adverse effects of the number of residual teeth on cognitive function among older adults.

## 1. Introduction

In aging populations, an increasing number of older adults are living with cognitive impairments. The pooled prevalence of cognitive impairment among Chinese older adults was 15.4% in 2021 [1]. Among older adults living in rural areas, the prevalence of cognitive impairment was 26.48% [2], with a higher prevalence reported in western China [3]. Moreover, cognitive impairment imposes an enormous disease burden [4]. Identifying the risk factors of cognitive impairment and early interventions can improve the quality of life of older adults and their caregivers.

An increasing number of studies have suggested that tooth loss in later life could increase the risk of cognitive impairment in older adults [5,6,7]. Studies have indicated that tooth loss could be an early indicator of accelerated aging [8,9]. A meta-analysis study [7] reported that older adults with more teeth had a decreased risk of dementia by approximately 50%. Another meta-analysis study [6] which examined longitudinal studies in Asian countries, also indicated that tooth loss is a risk factor for dementia.

However, the underlying mechanisms of the relationship between the number of residual teeth and cognitive function in older adults is still unclear. Studies have suggested that nutritional status plays an explanatory role in this relationship. More specifically, impaired dentition status is associated with poor dietary intake and malnutrition [10]. Malnutrition risks were associated with worse cognitive function [11]. Some studies suggested that numerous nutrients affect cognitive function [12,13]. Currently, only a limited-scale study [14] has examined nutrition as a mediating factor when investigating the relationship between the number of teeth, denture wearing, and cognition. Further verifications of this relationship are needed with studies that use larger samples. Consequently, we hypothesize that the association between the number of residual teeth and cognitive function is possibly mediated by nutritional pathways among 6634 multi-ethnic older adults from western China.

## 2. Materials and Methods

### 2.1. Study Protocol and Population

**Figure 1:**
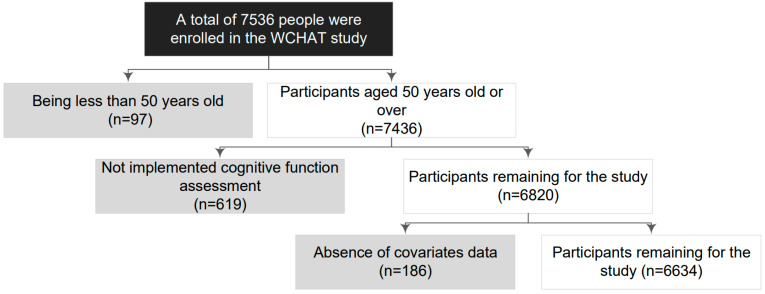
The flowchart of study participants; 6634 participants were analyzed in this study. Among those who were excluded from the study, 97 participants were less than 50 years old, 619 participants presented without cognition data, and 186 participants presented without covariates data.

The data in this cross-sectional study were obtained from a West China Health and Aging Trend (WCHAT) multicenter cohort study which focused on aging and health status in older adults. The cohort collected baseline data from July to December 2018, and the study was conducted in four provinces in western China, including Yunnan, Guizhou, Sichuan, and Xinjiang. Considering the topographical characteristics and scattered living patterns in different ethnic regions in western China, the county’s medical institution was selected as the primary venue for conducting the study. Ethics approval for this research was granted by the West China Hospital of Sichuan University (reference: 2017-445) on 31 January 2018. The register ID is 1800018895 in the Chinese Clinical Trial Registry. Informed consent was obtained from all respondents. Details of the protocol have been reported elsewhere [15]. The researchers, who majored in geriatrics from Sichuan University, completed comprehensive questionnaires to record older adults’ demographic characteristics and health-related data. All researchers were trained for 2 days in how to collect questionnaire data via face to face and one-on-one personal interviews. The interrater reliability achieved 90% through practical training on questionnaires. The questionnaire underwent cultural adaptations and preliminary testing to ensure its relevance to the specific cultural, lifestyle, and linguistic characteristics of western China. To be eligible to participate, the following inclusion criteria were established: (1) willingness to participate and to have provided signed informed consent, and (2) to be 50 years or older. The exclusion criteria were as follows: (1) absence of cognitive function assessment, and (2) lack of covariate data. From an initial sample of 7536 participants, 902 were excluded after applying the inclusion and exclusion criteria, and a total of 6634 participants were included in the study (Figure 1).

### 2.2. Data Collection

The number of residual teeth was calculated via a specialized oral examination. Cognitive function was assessed using the Chinese version of the 10-item Short Portable Mental Status Questionnaire (SPMSQ), which evaluates memory, orientation to time and place, current event information, and calculation. Higher error rates indicate more severe cognitive impairment. Pfeiffer’s criteria [16] classify cognitive function as follows: normal cognition (0–2), mild cognitive impairment (3–4), and moderate and severe cognitive impairment (5 or higher). This scoring adjusts for the biasing effects of educational background. The nutrition status was estimated by the Mini Nutritional Assessment-Short Form (MNA-SF). The MNA-SF is a reliable tool for distinguishing older adults who are malnourished or at risk of malnutrition. MNA-SF scores are categorized as follows: normal nutrition (12–14), at risk of malnutrition (8–11), and malnutrition (0–7) [17].

Additional sociodemographic covariates were age, sex, educational level, occupation, spouse status, longevity of family members, and ethnic groups (Han, Tibetan, Qiang, Yi, Uyghur, and others). “Others” refers to a few ethnic groups combined with the Zhuang, Manchu, Hui, Mongolian, and Tujia groups; they are classified as such due to the number of participants being less than 200. Basic lifestyle information was also collected: type of drinking water and history of smoking, alcohol use, and drinking tea.

### 2.3. Statistical Analysis

Descriptive data were summarized using proportions for categorical data and means with standard deviation (SD) for continuous data. Differences between the categories of cognitive impairment and the variables studied were analyzed using one-way ANOVA for continuous variables and the chi-squared test for categorical variables. Multiple comparisons were conducted using independent sample t-tests and chi-square tests. The Bonferroni correction was used to correct *p*-values for multiple comparisons.

The association between the number of residual teeth, MNA-SF scores, and cognitive scores was analyzed using linear regression, which used three separate models. We entered the number of residual teeth as a predictor variable, and cognitive scores as outcome variables, using two separate models: Model 1 was adjusted in accordance with age, sex, ethnic group, educational level, occupation, spouse status, longevity of family members, denture usage, and lifestyles (type of drinking water, history of smoking, alcohol use, and drinking tea); Model 2 was adjusted using Model 1 and the MNA-SF score. We entered the number of residual teeth as a predictor variable and MNA-SF scores as outcome variables in accordance with Model 3, which was adjusted using the same confounding factors as Model 1. R 4.1.3 was used to perform all statistical analyses in this study. We used the mediation R package to evaluate the influence of the MNA-SF score on the relationship between the number of residual teeth and cognitive scores. The path analysis was conducted (lavaan package) using the structural equation model (SEM) framework. The results were statistically significant at *p* < 0.05, and all *p* values were two-sided.

## 3. Results

The descriptive analyses of demographic characteristics and lifestyles were stratified in accordance with level of cognitive impairment, and they are summarized in Table 1. The participants had an average age of 62.39 (8.26) years, they were predominantly female (62.5%), and were ethnically Han (36.1%). The average number of residual teeth was 21.20 (9.52). The percentage of participants wearing a denture was 38%. The mean MNA-SF score was 12.58 (1.58). Participants with cognitive impairments had fewer residual teeth (21.83 [9.19] vs. 18.37 [10.19] vs. 16.03 [10.98], *p* < 0.001) and lower MNA-SF scores (12.86 [1.37] vs. 11.48 [1.6] vs. 10.03 [1.81], *p* < 0.001) than normal participants. The participants with cognitive impairments tended to be at risk of losing teeth and malnutrition. The Supplementary Material Table S1 showed the results of the multiple comparisons among the three groups.

Multiple linear regression demonstrated a significant negative association between the number of residual teeth and cognitive impairment (Model 1 *β* = −0.150; 95% CI: −0.190 to −0.111) (Table 2), and this association was independent of covariates. However, the coefficient of the association between the number of residual teeth and cognitive impairment decreased when the nutrition score was included as a parameter (Model 2 *β* = −0.089; 95% CI: −0.126 to −0.052). Moreover, the number of residual teeth was significantly associated with nutritional status (Model 3 *β* = 0.197; 95% CI: 0.153 to 0.241). This suggested that nutritional status may partly influence the association between the number of residual teeth and cognitive impairment.

**Figure 2:**
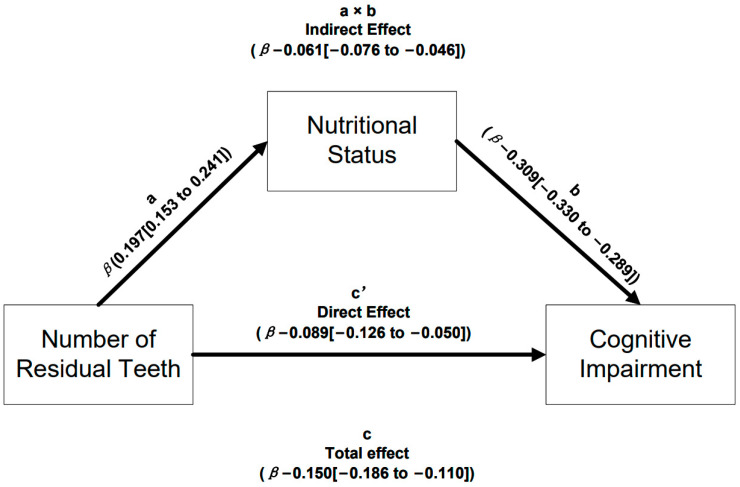
The mediating effect of nutritional status in the mediation model chart.

Mediation model analysis was performed to determine if nutritional status was the mediator of the effects regarding the number of residual teeth and cognitive impairment. As shown in Figure 2, mediation model analysis showed that a decline in the number of residual teeth was correlated with worse nutritional status (*β* = 0.197; 95% CI: 0.153 to 0.241); the nutritional status score showed a significant negative association with cognitive impairment (*β* = −0.309; 95% CI: −0.330 to −0.289). The mediating effects of the number of residual teeth on cognitive impairment, mediated by nutritional status, were statistically significant (*β* = −0.061; 95% CI: −0.076 to −0.046), and the direct effects were also statistically significant (*β* = −0.089; 95% CI: −0.126 to −0.050). The total effect of the number of residual teeth on cognitive impairment was statistically significant (*β* = −0.150; 95% CI: −0.186 to −0.110). This suggested that nutritional status partly mediates the association between cognitive impairment and the number of residual teeth. Expressed as a percentage, the effect of the number of residual teeth on cognitive impairment, mediated by nutritional status, was 40.66% (95% CI: 30.00% to 55.44%) (Supplementary Material Table S2). The mediation analysis’ statistical details were adjusted in accordance with the covariates in Table S2.

**Figure 3:**
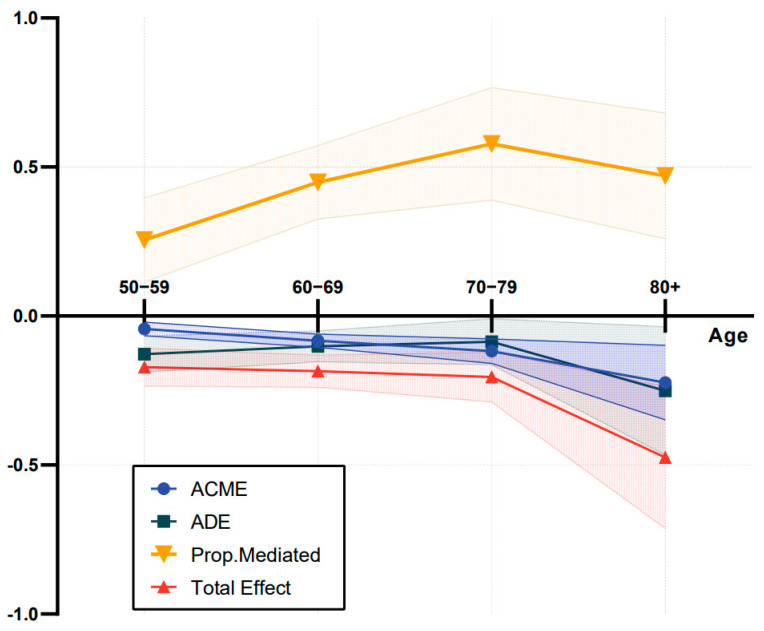
The line chart of mediation analysis in older adults stratified by age. Abbreviations. ACME: average causal mediation effects (indirect effects); ADE: average direct effects. Prop: proportion.

We conducted an additional mediation analysis by stratifying the sample into four subgroups based on age (Figure 3 and Supplementary Material Table S3). Among older adults in the four groups, negative correlations were identified with the indirect effects that were mediated through nutritional status, as well as the direct and total effects that the number of residual teeth have on cognitive impairment. Interestingly, expressed as a percentage, the mediating effect of nutritional status became more potent with age among individuals aged 50 to 79 years (50–59 group: 25.48% (95% CI: 11.37% to 43.35%); 60–69 group: 44.87% (95% CI: 32.56% to 64.93%); 70–79 group: 57.77% (95% CI: 38.89% to 93.50%)). However, in the subgroup of elderly individuals aged 80 years and older, the mediating effect became weaker (46.99% (95% CI: 25.89% to 84.36%)).

**Figure 4:**
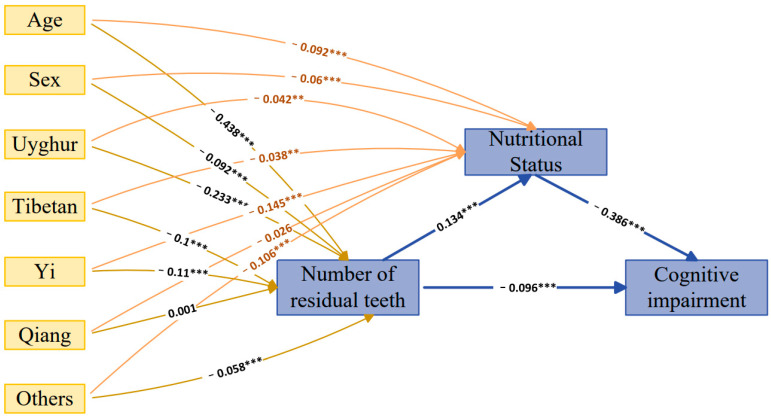
A pathway analysis using the SEM framework. This framework shows that the estimated coefficient of the number of residual teeth to cognitive impairment was −0.096, thus, it exhibited a negative influence. The SEM coefficient of the number of residual teeth to nutrition status was 0.134, thus, it exhibited a positive influence. The SEM coefficient of nutrition status to cognitive impairment was −0.386, thus, it exhibited a negative influence. Age, sex, and ethnic groups have different standardized coefficients to the number of the residual teeth and nutritional status. The *p* values of all the pathways shown in the framework were significant except for the Qiang groups (** *p* < 0.005; *** *p* < 0.001).

The SEM framework was used for path analysis (Figure 4). The correlations between the number of residual teeth and cognitive impairment, as well as nutritional status and cognitive impairment, were both negative. (SEM standardized co-efficient: −0.096 and −0.386). The correlation between the number of residual teeth and nutritional status was positive (SEM standardized co-efficient: 0.134). Furthermore, age, sex, and ethnic groups showed different estimate coefficients compared with the number of residual teeth and nutritional status. In particular, Uyghur groups showed the most obvious coefficients, with regard to the number of residual teeth, of all the ethnic groups (SEM standardized co-efficient: −0.233). Moreover, the strongest coefficients between ethnicity and nutritional status occurred in the Yi group (SEM standardized co-efficient: −0.145). The *p* value of the entire SEM pathway was statistically significant except for the *p* value of the Qiang groups (Figure 4 and Supplementary Material Table S4). These results further confirmed the association between the number of residual teeth, nutritional status, and cognitive function.

## 4. Discussion

Based on this cross-sectional study of 6634 older adults from multi-ethnic groups in western China, we identified the partial mediating role of nutritional status in the relationship between the number of residual teeth and cognitive impairment.

The mediation model in this study established a pathway from the number of residual teeth to cognitive impairment, partially mediated by nutritional status. This result aligns well with previous studies. A prior study [14] found that the mediating effect of nutritional status, expressed as a percentage, on the number of teeth, denture wearing, and severe cognitive impairment, was 23.1%. In this study, the mediating effect in older adults in western China, expressed as a percentage, was 40.66%; this was greater than previous results. The differences in cognitive assessment methods may have impacted the results of this study. In our study, the SPMSQ utilized was more sensitive than the Clinical Dementia Rating (CDR), particularly when screening mild cognitive impairment among older adults [18,19]. Therefore, the number of older adults that were identified as having mild cognitive impairments in this study is larger compared with previous studies. We speculated that nutrition may have a stronger influence on individuals with milder cognitive impairments, and thus, their cognitive function.

This study demonstrated a positive correlation between a greater number of remaining teeth, better nutritional status, and improved cognitive function in older adults. We believe that this mediation model consolidates and validates the perspectives of previous studies. First, tooth loss causes profound damage to the acquisition and utilization of nutrients. Older adults without teeth prefer soft food and they consume greater quantities of sugar [10,20] instead of foods such as fresh fruits and vegetables [21]. Yoshihara et al. [22] also suggested that the number of residual teeth was related to the intake of several nutrients; in particular, total protein, sodium, vitamin, and niacin. Second, suboptimal nutritional status impaired cognitive function. Some studies suggested that adherence to a healthy diet pattern, such as a Mediterranean diet, is associated with a lower risk of cognitive impairment [23,24]. This is attributed to the presence of several neuro-nutritional components in the diet [25]. For example, the consumption of omega-3 fatty acids from fish and plant sources can enhance cognitive health and mental health [26,27].

We revealed that the association between the number of residual teeth and cognitive impairment is mediated through nutritional status; this association was strengthened with increasing age. This finding might be attributed to the rate of tooth loss, which increased with age. Previous studies indicated that age is a significant predictor of tooth loss [28]. Older adults with 10 or fewer teeth find it difficult to meet dietary recommendations, and the number of such individuals has increased [29]. Therefore, we inferred that the substantial loss of teeth in relatively senior older adults may exacerbate nutritional issues and make cognitive impairments more severe. However, the mediating effect was reduced among older adults aged 80 years and older. This could be due to the limited representation of individuals in this age group, resulting in increased variability and larger errors in the analysis.

This study also found direct effects between the number of residual teeth and cognitive function. We considered that a higher number of residual teeth is directly related to enhanced cognitive performance to some extent. This finding is consistent with prior studies. Patients suffering from Alzheimer’s disease are characterized by a lower number of residual teeth [30]. Yusuke [31] also found that participants with some natural teeth had a 0.40 point higher cognitive function score than those with complete tooth loss. We assumed that this direct correlation may be due to genetic regulation in humans. Genetic factors affect the number of tooth phenotypes [32] and cognitive function [33]. For example, the matrix Gla protein gene (MGP), as a candidate gene, is implicated in the pathogenesis of tooth loss and cognitive impairment through the encoding of the expressed vitamin K binding protein [34,35,36].

Another interesting finding of our study is that ethnic groups had different effects on the number of residual teeth among older adults in western China, particularly in Uyghurs. Currently, there is insufficient research available to provide an explanation for this result. In previous studies, researchers reported that variations caused by genetic factors could influence the number of residual teeth and tooth anatomy [34]. Tan et al. [37] found that the EDARV370A genetic variant in Uyghurs played a key role in dental morphology. Therefore, we speculated that this genetic variation is one of the risk factors for the reduced number of residual teeth in Uygur elderly individuals compared with other ethnic groups. Further research is required to investigate additional factors (such as economic status, occupation, and lifestyle habits) that may influence the number of residual teeth. Furthermore, this study found that nutritional status produced different effects on various ethnic groups, with the Yi group exhibiting the poorest nutritional status. This result can be attributed to the relatively primitive lifestyle and unique dietary culture and customs maintained by the aboriginal Yi ethnic group. Yao et al. [38] reported that the prevalence of underweight people was 9.5% in the Yi Autonomous Prefecture. Dietary nutrient intakes of adult Yi people were varied, and there were differences between rural and urban areas [39]. Based on the abovementioned characteristics of ethnic groups, individualized assessments of health conditions are needed for minority ethnic older adults. Caregivers should implement targeted interventions that are tailored to meet their specific needs.

This study has several limitations that need to be acknowledged. Firstly, the absence of comprehensive oral health data, such as odontalgia, swallowing, and chewing abilities, may introduce potential bias. Additionally, the data on cognitive function and nutritional status were collected by self-report questionnaires, which could be subject to recall bias and may not fully capture the true status of the participants. Therefore, it is necessary to be careful when interpreting the results. Future studies should aim to comprehensively evaluate nutritional status, oral health, and cognitive function using objective measures, and this mediation model should be validated in a longitudinal study.

## 5. Conclusions

The mediation model in this study established a pathway from the number of residual teeth to cognitive impairment, partially mediated by nutritional status. This finding indicated that enhancing the nutrition of older adults could reduce the adverse effects of the number of residual teeth on cognitive function among older adults in Western China. The findings could provide guidance for caregivers when providing effective strategies to preserve cognitive function in older adults with few residual teeth. Timely nutrition interventions can help prevent degradations in cognitive function.

## Figures and Tables

**Table 1:** Sample characteristics stratified in accordance with cognitive impairment.

Characters	All n = 6634	Normal n = 5614	Mild Cognitive Impairment n = 733	Moderate to Severe Cognitive Impairment n = 287	*p* Value
**Age, mean (SD) ^abc^**	62.39 (8.26)	61.95 (8.05)	64.13 (8.75)	66.47 (9.37)	<0.001
**Age group, n (%) ^abc^**					<0.001
50–59	2633 (39.7%)	2306 (87.6%)	254 (9.6%)	73 (2.8%)	
60–69	2643 (39.8%)	2279 (86.2%)	266 (10.1%)	98 (3.7%)	
70–79	1174 (17.7%)	906 (77.2%)	176 (15%)	92 (7.8%)	
80+	184 (2.8%)	123 (66.8%)	37 (20.1%)	24 (13%)	
**Sex, n (%) ^abc^**					<0.001
Male	2487 (37.5%)	2261 (90.9%)	178 (7.2%)	48 (1.9%)	
Female	4147 (62.5%)	3353 (80.9%)	555 (13.4%)	239 (5.8%)	
**Ethnic group, n (%) ^abc^**					<0.001
Han	2394 (36.1%)	2168 (90.6%)	175 (7.3%)	51 (2.1%)	
Tibetan	1276 (19.2%)	1035 (81.1%)	171 (13.4%)	70 (5.5%)	
Qiang	1272 (19.2%)	1077 (84.7%)	154 (12.1%)	41 (3.2%)	
Yi	607 (9.1%)	433 (71.3%)	103 (17%)	71 (11.7%)	
Uyghur	563 (8.5%)	483 (85.8%)	68 (12.1%)	12 (2.1%)	
Other	522 (7.9%)	418 (80.1%)	62 (11.9%)	42 (8%)	
**Educational level, n (%) ^abc^**					<0.001
No formal education	1842 (27.8%)	1266 (68.7%)	375 (20.4%)	201 (10.9%)	
Elementary school	2237 (33.7%)	1990 (89%)	194 (8.7%)	53 (2.4%)	
Middle school	1438 (21.7%)	1349 (93.8%)	74 (5.1%)	15 (1%)	
High school and above	1117 (16.8%)	1009 (90.3%)	90 (8.1%)	18 (1.6%)	
**Occupation, n (%) ^abc^**					<0.001
Farmer	4321 (65.1%)	3493 (80.8%)	576 (13.3%)	252 (5.8%)	
Worker	531 (8%)	504 (94.9%)	23 (4.3%)	4 (0.8%)	
White-collar	696 (10.5%)	632 (90.8%)	54 (7.8%)	10 (1.4%)	
Others	1086 (16.4%)	985 (90.7%)	80 (7.4%)	21 (1.9%)	
**Spouse status, n (%) ^abc^**					<0.001
Without spouse	1098 (16.6%)	846 (77%)	158 (14.4%)	94 (8.6%)	
With Spouse	5536 (83.4%)	4768 (86.1%)	575 (10.4%)	193 (3.5%)	
**Longevity of families, n (%) ^ab^**					<0.001
No	5545 (83.6%)	4630 (83.5%)	650 (11.7%)	265 (4.8%)	
Yes	1089 (16.4%)	984 (90.4%)	83 (7.6%)	22 (2%)	
**Number of residual teeth, mean (SD) ^abc^**	21.20 (9.52)	21.83 (9.19)	18.37 (10.19)	16.03 (10.98)	<0.001
**Number of residual teeth, n (%) ^abc^**					<0.001
0–8	993 (15%)	742 (74.7%)	165 (16.6%)	86 (8.7%)	
9–16	724 (10.9%)	560 (77.3%)	109 (15.1%)	55 (7.6%)	
17–24	1512 (22.8%)	1279 (84.6%)	182 (12%)	51 (3.4%)	
25–32	3405 (51.3%)	3033 (89.1%)	277 (8.1%)	95 (2.8%)	
**Denture usage, n (%) ^a^**					0.027
No	4112 (62%)	3517 (85.5%)	423 (10.3%)	172 (4.2%)	
Yes	2522 (38%)	2097 (83.1%)	310 (12.3%)	115 (4.6%)	
**MNA-SF score, mean (SD) ^abc^**	12.58 (1.58)	12.86 (1.37)	11.48 (1.6)	10.03 (1.81)	<0.001
**MNA-SF assessment status, n (%) ^abc^**					<0.001
Normal	5185 (78.2%)	4697 (90.6%)	408 (7.9%)	80 (1.5%)	
Nutrition risk	1393 (21%)	907 (65.1%)	310 (22.3%)	176 (12.6%)	
Malnutrition	56 (0.8%)	10 (17.9%)	15 (26.8%)	31 (55.4%)	
**Type of drinking water, n (%) ^b^**					0.004
Tap water	5318 (80.2%)	4530 (85.2%)	574 (10.8%)	214 (4%)	
Well water	410 (6.2%)	348 (84.9%)	48 (11.7%)	14 (3.4%)	
Spring water	716 (10.8%)	574 (80.2%)	91 (12.7%)	51 (7.1%)	
Others	190 (2.9%)	162 (85.3%)	20 (10.5%)	8 (4.2%)	
**History of smoking, n (%) ^ab^**					<0.001
No	5353 (80.7%)	4464 (83.4%)	635 (11.9%)	254 (4.7%)	
Yes	1281 (19.3%)	1150 (89.8%)	98 (7.7%)	33 (2.6%)	
**History of alcohol use, n (%) ^ab^**					<0.001
No	4911 (74%)	4091 (83.3%)	582 (11.9%)	238 (4.8%)	
Yes	1723 (26%)	1523 (88.4%)	151 (8.8%)	49 (2.8%)	
**History of drinking tea, n (%) ^ab^**					<0.001
No	3687 (55.6%)	3019 (81.9%)	467 (12.7%)	201 (5.5%)	
Yes	2947 (44.4%)	2595 (88.1%)	266 (9%)	86 (2.9%)	

^a^ The multiple comparisons revealed significant differences between participants with mild cognitive impairments and those with normal cognitive function. ^b^ The significant differences between participants with moderate to severe cognitive impairments and those with normal cognitive function. ^c^ The significant differences between participants with moderate to severe cognitive impairments and those with mild cognitive impairments.

**Table 2:** Multiple linear regression analysis of cognitive function, the number of residual teeth, and nutritional status in older adults.

Outcome Variable		Model 1: Cognitive Score		Model 2: Cognitive Score		Model 3: MNA-SF Score	
		*β*	95% CI ^1^	*β*	95% CI	*β*	95% CI
**MNA-SF Score**		--	--	−0.309	−0.330 to −0.289	--	--
**Number of Residual Teeth**		−0.150	−0.190 to −0.111	−0.089	−0.126 to −0.052	0.197	0.153 to 0.241
**Age group**	50–59	Ref ^2^		Ref		Ref	
	60–69	0.007	−0.070 to 0.085	0.021	−0.052 to 0.094	0.043	−0.043 to 0.130
	70–79	0.338	0.233 to 0.444	0.256	0.156 to 0.355	−0.268	−0.386 to −0.15
	80+	0.598	0.381 to 0.815	0.456	0.252 to 0.66	−0.461	−0.704 to −0.219
**Sex**	Male	Ref ^2^		Ref		Ref	
	Female	0.414	0.324 to 0.504	0.378	0.293 to 0.462	−0.116	−0.217 to −0.016
**Ethnic group**	Han	Ref		Ref		Ref	
	Tibetan	0.432	0.332 to 0.532	0.382	0.288 to 0.476	−0.163	−0.275 to −0.051
	Qiang	0.157	0.060 to 0.254	0.145	0.054 to 0.235	−0.039	−0.148 to 0.069
	Yi	0.809	0.683 to 0.935	0.610	0.491 to 0.729	−0.643	−0.784 to −0.503
	Uyghur	0.335	0.199 to 0.472	0.222	0.094 to 0.35	−0.366	−0.518 to −0.213
	Others	0.431	0.301 to 0.561	0.258	0.135 to 0.381	−0.560	−0.706 to −0.415
**Educational level**	No formal education	Ref		Ref		Ref	
	Elementary school	−0.844	−0.930 to −0.757	−0.714	−0.795 to −0.632	0.421	0.325 to 0.517
	Middle school	−0.754	−0.857 to −0.650	−0.612	−0.709 to −0.514	0.458	0.343 to 0.574
	High school and above	0.129	−0.005 to 0.263	0.226	0.100 to 0.351	0.312	0.163 to 0.462
**Occupation**	Farmer	Ref		Ref		Ref	
	Worker	−0.233	−0.362 to −0.105	−0.222	−0.342 to −0.102	0.037	−0.106 to 0.180
	White-collar	−0.271	−0.413 to −0.130	−0.197	−0.330 to −0.065	0.240	0.082 to 0.398
	Others	−0.196	−0.295 to −0.098	−0.152	−0.244 to −0.059	0.143	0.033 to 0.253
**Spouse status**	Without spouse	Ref		Ref		Ref	
	With spouse	−0.053	−0.146 to 0.040	−0.024	−0.111 to 0.063	0.095	−0.009 to 0.198
**Longevity of family**	No	Ref		Ref		Ref	
	Yes	−0.162	−0.251 to −0.073	−0.147	−0.231 to −0.063	0.048	−0.052 to 0.147
**Denture usage**	No	Ref		Ref		Ref	
	Yes	−0.200	−0.282 to −0.118	−0.152	−0.228 to −0.075	−0.2	−0.282 to −0.118
**Type of drinking water**	Tap water	Ref		Ref		Ref	
	Well water	−0.008	−0.146 to 0.13	0.002	−0.128 to 0.131	0.031	−0.123 to 0.186
	Spring water	0.153	0.044 to 0.262	0.118	0.016 to 0.220	−0.113	−0.235 to 0.009
	Others	−0.062	−0.260 to 0.136	−0.019	−0.205 to 0.167	0.138	−0.083 to 0.359
**History of smoking**	No	Ref		Ref		Ref	
	Yes	0.071	−0.034 to 0.175	−0.001	−0.099 to 0.098	−0.230	−0.347 to −0.113
**History of alcohol use**	No	Ref		Ref		Ref	
	Yes	0.014	−0.073 to 0.100	0.030	−0.051 to 0.111	0.054	−0.043 to 0.150
**History of drinking tea**	No	Ref		Ref		Ref	
	Yes	−0.114	−0.187 to −0.042	−0.056	−0.124 to 0.012	0.189	0.109 to 0.270
**Intercept**		1.679	1.459 to 1.899	5.306	4.991 to 5.621	11.722	11.476 to 11.967
**Observations**		6634		6634		6634	
**R^2^**		0.183		0.280		0.096	
**Adjusted R^2^**		0.180		0.278		0.092	
**Residual standard error**		1.349		1.266		1.51	
**F Statistic ([df ^3^]; *p* value)**		59.12 ([25, 6608]; <0.001)		98.98 ([26, 6607]; <0.001)		27.91 ([25, 6608]; <0.001)	

^1^ Cl: confidence interval; ^2^ Ref: reference group, ^3^ df: degree of freedom; The association between residual teeth and cognitive function was revealed in Model 1, and was adjusted using the MNA-SF score in Model 2. Model 3 showed the linear association between the MNA-SF score and number of residual teeth. The remaining covariates (including age, sex, ethnic group, occupation, educational level, spouse status, longevity of family members, denture status, type of drinking water, and history of smoking, alcohol use, and drinking tea) were adjusted in all models.

## Data Availability

The data that support the findings of this study are available from the corresponding author, upon reasonable request.

## Supplementary Materials

The following supporting information can be downloaded at: https://www.mdpi.com/article/10.3390/nu15143089/s1, Table S1: The results of multiple comparisons and the Bonferroni correction; Table S2: MNA-SF score as a mediator of the effect of residual teeth on cognitive function; Table S3: Mediating analysis in older adults stratified by age; Table S4: The summary of the structural equation model.

## References

[1] Deng Y., Zhao S., Cheng G., Yang J., Li B., Xu K., Xiao P., Li W., Rong S. (2021). The Prevalence of Mild Cognitive Impairment among Chinese People: A Meta-Analysis. Neuroepidemiology.

[2] Cong L., Ren Y., Wang Y., Hou T., Dong Y., Han X., Yin L., Zhang Q., Feng J., Wang L. (2023). Mild Cognitive Impairment among Rural-Dwelling Older Adults in China: A Community-Based Study. Alzheimer’s Dement..

[3] Xue J., Li J., Liang J., Chen S. (2018). The Prevalence of Mild Cognitive Impairment in China: A Systematic Review. Aging Dis..

[4] Tahami Monfared A.A., Byrnes M.J., White L.A., Zhang Q. (2022). The Humanistic and Economic Burden of Alzheimer’s Disease. Neurol. Ther..

[5] Kamer A.R., Craig R.G., Dasanayake A.P., Brys M., Glodzik-Sobanska L., de Leon M.J. (2008). Inflammation and Alzheimer’s Disease: Possible Role of Periodontal Diseases. Alzheimer’s Dement..

[6] Shen T., Lv J., Wang L., Wang W., Zhang D. (2016). Association between Tooth Loss and Dementia among Older People: A Meta-Analysis. Int. J. Geriatr. Psychiatry.

[7] Oh B., Han D.-H., Han K.-T., Liu X., Ukken J., Chang C., Dounis K., Yoo J.W. (2018). Association between Residual Teeth Number in Later Life and Incidence of Dementia: A Systematic Review and Meta-Analysis. BMC Geriatr..

[8] Dibello V., Zupo R., Sardone R., Lozupone M., Castellana F., Dibello A., Daniele A., De Pergola G., Bortone I., Lampignano L. (2021). Oral Frailty and Its Determinants in Older Age: A Systematic Review. Lancet Healthy Longev..

[9] Avlund K., Schultz-Larsen K., Christiansen N., Holm-Pedersen P. (2011). Number of Teeth and Fatigue in Older Adults: Teeth and Fatigue. J. Am. Geriatr. Soc..

[10] Hung H.-C., Willett W., Ascherio A., Rosner B.A., Rimm E., Joshipura K.J. (2003). Tooth Loss and Dietary Intake. J. Am. Dent. Assoc..

[11] Katsas K., Mamalaki E., Kontogianni M.D., Anastasiou C.A., Kosmidis M.H., Varlamis I., Hadjigeorgiou G.M., Dardiotis E., Sakka P., Scarmeas N. (2020). Malnutrition in Older Adults: Correlations with Social, Diet-Related, and Neuropsychological Factors. Nutrition.

[12] Scarmeas N., Anastasiou C.A., Yannakoulia M. (2018). Nutrition and Prevention of Cognitive Impairment. Lancet Neurol..

[13] Lu Z., He R., Zhang Y., Li B., Li F., Fu Y., Rong S. (2023). Relationship between Whole-Blood Magnesium and Cognitive Performance among Chinese Adults. Nutrients.

[14] Suma S., Furuta M., Takeuchi K., Tomioka M., Iwasa Y., Yamashita Y. (2022). Number of Teeth, Denture Wearing and Cognitive Function in Relation to Nutritional Status in Residents of Nursing Homes. Gerodontology.

[15] Hou L., Liu X., Zhang Y., Zhao W., Xia X., Chen X., Lin X., Yue J., Ge N., Dong B. (2021). Cohort Profile: West China Health and Aging Trend (WCHAT). J. Nutr. Health Aging.

[16] Pfeiffer E. (1975). A Short Portable Mental Status Questionnaire for the Assessment of Organic Brain Deficit in Elderly Patients. J. Am. Geriatr. Soc..

[17] Kaiser M.J., Bauer J.M., Ramsch C., Uter W., Guigoz Y., Cederholm T., Thomas D.R., Anthony P., Charlton K.E., Maggio M. (2009). Validation of the Mini Nutritional Assessment Short-Form (MNA^®^-SF): A Practical Tool for Identification of Nutritional Status. J. Nutr. Health Aging.

[18] Morris J.C. (1993). The Clinical Dementia Rating (CDR): Current Version and Scoring Rules. Neurology.

[19] Zhuang L., Yang Y., Gao J. (2021). Cognitive Assessment Tools for Mild Cognitive Impairment Screening. J. Neurol..

[20] Shen J., Qian S., Huang L., Tao Y., Chen H., Deng K., Yang F., Zong G., Zheng Y., Wang X. (2023). Association of the Number of Natural Teeth with Dietary Diversity and Nutritional Status in Older Adults: A Cross-Sectional Study in China. J. Clin. Periodontol..

[21] Komagamine Y., Kanazawa M., Iwaki M., Jo A., Suzuki H., Amagai N., Minakuchi S. (2016). Combined Effect of New Complete Dentures and Simple Dietary Advice on Nutritional Status in Edentulous Patients: Study Protocol for a Randomized Controlled Trial. Trials.

[22] Yoshihara A., Watanabe R., Nishimuta M., Hanada N., Miyazaki H. (2005). The Relationship between Dietary Intake and the Number of Teeth in Elderly Japanese Subjects. Gerodontology.

[23] Liu Y.-H., Gao X., Na M., Kris-Etherton P.M., Mitchell D.C., Jensen G.L. (2020). Dietary Pattern, Diet Quality, and Dementia: A Systematic Review and Meta-Analysis of Prospective Cohort Studies. J. Alzheimer’s Dis..

[24] Bhushan A., Fondell E., Ascherio A., Yuan C., Grodstein F., Willett W. (2018). Adherence to Mediterranean Diet and Subjective Cognitive Function in Men. Eur. J. Epidemiol..

[25] Badaeva A.V., Danilov A.B., Clayton P., Moskalev A.A., Karasev A.V., Tarasevich A.F., Vorobyeva Y.D., Novikov V.N. (2023). Perspectives on Neuronutrition in Prevention and Treatment of Neurological Disorders. Nutrients.

[26] Dighriri I.M., Alsubaie A.M., Hakami F.M., Hamithi D.M., Alshekh M.M., Khobrani F.A., Dalak F.E., Hakami A.A., Alsueaadi E.H., Alsaawi L.S. (2022). Effects of Omega-3 Polyunsaturated Fatty Acids on Brain Functions: A Systematic Review. Cureus.

[27] Morales-Suárez-Varela M., Amezcua-Prieto C., Llopis-Gonzalez A., Ayan Perez C., Mateos-Campos R., Hernández-Segura N., Ortiz-Moncada R., Almaraz A., Alguacil J., Delgado Rodríguez M. (2023). Prevalence of Depression and Fish Consumption among First Year Spanish University Students: UniHcos Project. Nutrients.

[28] Baelum V., Luan W.-M., Chen X., Fejerskov O. (1997). Predictors of Tooth Loss over 10 Years in Adult and Elderly Chinese. Community Dent. Oral Epidemiol..

[29] Savoca M.R., Arcury T.A., Leng X., Chen H., Bell R.A., Anderson A.M., Kohrman T., Frazier R.J., Gilbert G.H., Quandt S.A. (2010). Severe Tooth Loss in Older Adults as a Key Indicator of Compromised Dietary Quality. Public Health Nutr..

[30] Dioguardi M., Di Gioia G., Caloro G.A., Capocasale G., Zhurakivska K., Troiano G., Lo Russo L., Lo Muzio L. (2019). The Association between Tooth Loss and Alzheimer’s Disease: A Systematic Review with Meta-Analysis of Case Control Studies. Dent. J..

[31] Matsuyama Y. (2023). Time-Varying Exposure Analysis of the Relationship between Sustained Natural Dentition and Cognitive Decline. J. Clin. Periodontol..

[32] Khalaf K., Brook A.H., Smith R.N. (2022). Genetic, Epigenetic and Environmental Factors Influence the Phenotype of Tooth Number, Size and Shape: Anterior Maxillary Supernumeraries and the Morphology of Mandibular Incisors. Genes.

[33] Aimone J.B., Li Y., Lee S.W., Clemenson G.D., Deng W., Gage F.H. (2014). Regulation and Function of Adult Neurogenesis: From Genes to Cognition. Physiol. Rev..

[34] Hirano H., Ezura Y., Ishiyama N., Yamaguchi M., Nasu I., Yoshida H., Suzuki T., Hosoi T., Emi M. (2003). Association of Natural Tooth Loss with Genetic Variation at the Human Matrix Gla Protein Locus in Elderly Women. J. Hum. Genet..

[35] Kozioł-Kozakowska A., Maresz K. (2022). The Impact of Vitamin K2 (Menaquionones) in Children’s Health and Diseases: A Review of the Literature. Children.

[36] Shea M.K., Wang J., Barger K., Weiner D.E., Booth S.L., Seliger S.L., Anderson A.H., Deo R., Feldman H.I., Go A.S. (2022). Vitamin K Status and Cognitive Function in Adults with Chronic Kidney Disease: The Chronic Renal Insufficiency Cohort. Curr. Dev. Nutr..

[37] Tan J., Peng Q., Li J., Guan Y., Zhang L., Jiao Y., Yang Y., Wang S., Jin L. (2014). Characteristics of Dental Morphology in the Xinjiang Uyghurs and Correlation with the EDARV370A Variant. Sci. China Life Sci..

[38] Yao S., Wang J., Xiao S., Jin X., Xiong M., Peng J., Xu T. (2020). Inadequate Nutrition and Associated Factors in Children Aged 6 to 24 Months—4 Counties, Liangshan Yi Autonomous Prefecture, China, 2018. China CDC Wkly..

[39] Zhou J., Huang C., Xu Y., Sun G., Li X., Piao J., Yang X. (2003). The dietary patterns and nutritional status of adult Yi people in Liangshan autonomous region. Wei Sheng Yan Jiu.

