# Supplementary material for: The Mediating Effects of Nutritional Status on the Relationship between Number of Residual Teeth and Cognitive Function among Older Adults: A Cross-Sectional Multicenter Study
Source: Nutrients. 2023 Jul 10;15(14):3089. doi: 10.3390/nu15143089 (PMC10384863; doi:10.3390/nu15143089)
Supplement: Supplementary file 1 [file nutrients-15-03089-s001.zip › nutrients-2484293-SI.pdf]

Supplementary Materials

Table S1. The results of multiple comparisons and Bonferroni correction.

Characters	Multiple comparisons		
	Mild Cognitive Impairment vs. Normal	Moderate to Severe Cognitive Impairment vs. Normal	Moderate to Severe Cognitive Impairment vs. Mild Cognitive Impairment
**Age, mean (SD)**	<0.001	<0.001	<0.001
**Age group, n (%)**	<0.001	<0.001	0.007
**Sex, n (%)**	<0.001	<0.001	0.034
**Ethnic group, n (%)**	<0.001	<0.001	<0.001
**Educational level, n (%)**	<0.001	<0.001	<0.001
**Occupation, n (%)**	<0.001	<0.001	0.02
**Spouse status, n (%)**	<0.001	<0.001	<0.001
**Longevity families, n (%)**	<0.001	<0.001	0.32
**Number of residual teeth, mean (SD)**	<0.001	<0.001	0.006
**Number of residual teeth, n (%)**	<0.001	<0.001	0.016
**Denture usage, n (%)**	0.032	1	1
**MNA-SF score, mean (SD)**	<0.001	<0.001	<0.001
**MNA-SF assessment status, n (%)**	<0.001	<0.001	<0.001
**Type of drinking water, n (%)**	0.921	0.002	0.403
**History of smoking, n (%)**	<0.001	<0.001	1
**History of alcohol use, n (%)**	<0.001	<0.001	0.704
**History of drinking tea, n (%)**	<0.001	<0.001	0.199

Table S2. MNA-SF score as mediator of the effect of residual teeth on cognitive function.

		Model 1		Model 2	
		β	95% CI	β	95% CI
**MNA-SF score**	**ACME**	-0.099	-0.114 to -0.084	-0.061	-0.076 to -0.046
	**ADE**	-0.130	-0.164 to -0.097	-0.089	-0.126 to -0.050
	**Total Effect**	-0.229	-0.264 to -0.194	-0.150	-0.186 to -0.110
	**Proportion Mediated**	0.431	0.361 to 0.505	0.407	0.300 to 0.554

Mediation analysis statistic details were adjusted none covariate in Model 1, and were adjusted by denture usage, sex, age, ethnic groups, occupation, educational level, spouse status, longevity families, and life styles (type of drinking water, smoking, alcohol use and drinking tea) in Model 2. Abbreviations. ACME: average causal mediation effects (indirect effect); ADE: average direct effects; Cl: confidence Interval.

Table S3. The mediating analysis in older adults stratified by age.

Group	Parameter	β	95% Confidence Interval
50-59 years	ACME	-0.0439	-0.0672 to -0.019
	ADE	-0.1285	-0.1888 to -0.0675
	Total Effect	-0.1724	-0.2363 to -0.1067
	Proportion Mediated	0.2548	0.1137 to 0.4335
60-69 years	ACME	-0.0834	-0.1053 to -0.0621
	ADE	-0.102	-0.1538 to -0.0486
	Total Effect	-0.1854	-0.2394 to -0.1301
	Proportion Mediated	0.4487	0.3256 to 0.6493
70-79 years	ACME	-0.1184	-0.1598 to -0.0797
	ADE	-0.0865	-0.1639 to -0.0084
	Total Effect	-0.2049	-0.2893 to -0.1189
	Proportion Mediated	0.5777	0.3889 to 0.935
80+ years	ACME	-0.2239	-0.3493 to -0.1117
	ADE	-0.2517	-0.4672 to -0.0439
	Total Effect	-0.4756	-0.7147 to -0.2499
	Proportion Mediated	0.4699	0.2589 to 0.8436

Table S4. The summary of structural equation model.

Pathway	Estimate	SE	z	*P*	LLCI	ULCI	*β*
Nutritional Status <- Number of residual teeth	0.193	0.019	9.909	<0.001	0.154	0.231	0.134
Nutritional Status <- Sex	-0.196	0.039	-4.965	<0.001	-0.273	-0.118	-0.06
Nutritional Status <- Age	-0.018	0.003	-6.865	<0.001	-0.023	-0.013	-0.092
Nutritional Status <- Tibetan	-0.153	0.054	-2.85	0.004	-0.258	-0.048	-0.038
Nutritional Status <- Qiang	-0.103	0.053	-1.949	0.051	-0.207	0.001	-0.026
Nutritional Status <- Yi	-0.795	0.07	-11.356	<0.001	-0.932	-0.657	-0.145
Nutritional Status <- Uyghur	-0.238	0.074	-3.219	0.001	-0.383	-0.093	-0.042
Nutritional Status <- Others	-0.623	0.074	-8.422	<0.001	-0.768	-0.478	-0.106
Number of residual teeth <- Sex	-0.209	0.025	-8.453	<0.001	-0.258	-0.161	-0.092
Number of residual teeth <- Age	-0.058	0.001	-40.094	<0.001	-0.061	-0.056	-0.438
Number of residual teeth <- Tibetan	-0.279	0.034	-8.271	<0.001	-0.345	-0.213	-0.1
Number of residual teeth <- Qiang	0.002	0.033	0.061	0.952	-0.064	0.068	0.001
Number of residual teeth <- Yi	-0.421	0.044	-9.587	<0.001	-0.507	-0.335	-0.11
Number of residual teeth <- Uyghur	-0.918	0.045	-20.267	<0.001	-1.007	-0.83	-0.233
Number of residual teeth <- Others	-0.238	0.047	-5.107	<0.001	-0.33	-0.147	-0.058
Cognitive impairment <- Number of residual teeth	-0.13	0.015	-8.447	<0.001	-0.16	-0.1	-0.096
Cognitive impairment <- Nutritional Status	-0.363	0.011	-33.907	<0.001	-0.384	-0.342	-0.386

β is standardized coefficient.
